# Neurodevelopmental Disorders: From Pathophysiology to Novel Therapeutic Approaches

**DOI:** 10.3390/biomedicines10030623

**Published:** 2022-03-07

**Authors:** Markus Kosel, Nader Perroud

**Affiliations:** Division of Adult Psychiatry, Geneva Medical School, Geneva University Hospitals, University of Geneva, 1211 Geneva, Switzerland; nader.perroud@hcuge.ch

This special issue of Biomedicines on Neurodevelopmental Disorders (NDD): “From Pathophysiology to Novel Therapeutic Approaches”, is a precursor of what we hope will develop into a thriving and inspiring transdisciplinary field, including genetics, psychiatry, neurology, as well as basic and applied neurosciences and molecular biology in the research area. Indeed, there are, to date, not many comprehensive approaches to NDDs, especially in the clinical field. A remarkable exception is the inspiring opinion article by Thapar et al. [1]. Indeed, the clinical concept of NDD, as implemented by the Diagnostic and Statistical Manual, 5th edition, includes a wide range of heterogeneous disorders, such as intellectual disabilities, communication disorders, autism spectrum disorder (ASD), attention deficit/hyperactivity disorder (ADHD), specific learning disorder, and motor disorders [2].

As is usually the case, we focus on some specific disorders, mainly on ASD, ADHD and externalized symptoms from childhood to adulthood, based on the renewed appreciation of their longitudinal and whole-life perspective, proposed by the DMS-5. Basic aspects of the pathophysiology of these disorders and their treatments are covered.

In this special issue, a first review addresses the topic of externalized symptoms in adolescents and non-pharmaceutical therapeutic interventions. Constanty and colleagues [3] identified 239 studies, mainly conducted in North America, including 24,180 adolescents, from which about 13,341 received an intervention. They found that interventions integrating multiple approaches, targeting emotion, cognition and social domains were the most common type of interventions used. They were particularly used for aggressiveness, conduct problems, and delinquency. Some gaps were identified concerning the physiology of self-regulation processes, as well as promising tools, such as biofeedback, neurofeedback, and therapeutic programs, targeting neuropsychological processes (e.g., cognitive remediation). This review, thus, offers an interesting overview of current non-pharmaceutical interventions for externalized symptoms, with cues for researchers on the next step interventions. In a second review, Saccaro and colleagues [4] studied the link between Attention Deficit Hyperactivity Disorder (ADHD) and peripheral inflammation and stress. The authors discuss the existence of immune dysfunction in ADHD as possibly resulting from exposure to stress and anxiety, similarly to what is found in affective disorders. Although it is still difficult to establish the sequence of interactions of these various factors and to determine with certainty whether ADHD is the cause or the consequence of immune disorders, this review offers a new line of thought in the pathophysiology of ADHD and in the development of future therapeutic approaches, based on interventions targeting the immune system.

Three original articles address pathophysiological mechanisms of the following two neurodevelopmental disorders: ADHD and ASD. Latrèche and colleagues [5] used an eye-tracking technology to measure social orienting in 95 young children with ASD and 16 children with typical development. These children were asked to watch a 29-s video of a woman engaging in child-directed speech. The authors show that the level of attention directed to faces is not only associated with the level of autistic symptoms, cognitive and adaptive skills, but also that baseline measures predict treatment outcomes (unstructured interventions ranging from speech therapy and occupational therapy to multiple interventions). This study offers us a way to personalize interventions and to identify subgroups of ASD patients that may better benefit from intensive early intervention. Brunkhorst-Kanaan and colleagues [6] based their study on recent whole-genome sequencing studies, identifying genetic variations in genes involved in fatty acid metabolism and their association with ADHD. They report that ADHD patients have increased plasma concentrations of sphingosine-1-phosphate (S1P d18:1), sphinganine-1-phosphate (S1P d18:0), endocannabinoids, anandamide (AEA) and arachidonoylglycerol, compared to controls and/or patients with affective disorders. This study provides us with new insight in the pathophysiology of ADHD, opening a new way to improve the diagnosis of ADHD, based on plasma levels of these lipid species. Rüfenacht and colleagues [7], based on a sample of 470 French-speaking outpatients (N = 279 ADHD, N = 70 BPD, N = 60 ADHD + BPD, N = 61 clinical controls) emphasize the importance of early life adversities in the aetiology of emotion dysregulation in ADHD. They also highlight the mediating effect of insecure attachment in the relationship between history of childhood maltreatment and emotion reactivity and the use of non-adaptive cognitive emotion regulation strategies in adult ADHD.

Our understanding of basic aspects of the pathophysiology of neurodevelopmental disorders and efficient therapies is still incomplete and the adult population suffering from these disorders is especially widely under-researched and rarely benefits from adequate treatments. However, this special issue of Biomedicines opens new promising perspectives, not only from the pathophysiological point of view, highlighting the main role played by stress and inflammatory processes, as well as metabolic pathways in the aetiology disorders, such as ADHD, but also by suggesting new clinical tools, such as neurofeedback or biofeedback, for the treatment of externalized symptoms in children, or even the use of eye-tracking technology to personalize treatment for ASD patients. Researchers and clinicians should, thus, benefit from these new technologies to better target their interventions in the future.

## Data Availability

Not applicable.
